# Photon-counting computed tomography in pancreatic ductal adenocarcinoma: correlation of iodine concentration with histopathological and clinical parameters

**DOI:** 10.1186/s12880-026-02980-2

**Published:** 2026-11-04

**Authors:** Saher Saeed, Jonas Helbing, Julius Niehoff, Nina Pauline Haag, Maria Katz, Ramona Muecke, Marwin-Jonathan Saehn, Saleem Elhabash, Sven Arke Lang, Christoph Moenninghoff, Jan Robert Kroeger, Jan Borggrefe, Alexey Surov, Matthias Michael Woeltjen

**Affiliations:** 1 Ruhr University Bochum, Department of Radiology, Neuroradiology and Nuclear Medicine, Johannes Wesling Hospital, Minden, Germany; 2 Faculty of Medicine, Ruhr University Bochum, Bochum, Germany; 3 Department of General Surgery, Visceral, Thoracic and Endocrine Surgery, Johannes Wesling Hospital, Minden, Germany

**Keywords:** Photon-counting computed tomography, Pancreatic ductal adenocarcinoma, Iodine concentration, Iodine maps, CA 19-9, Quantitative imaging, Spectral CT, Ki-67

## Abstract

**Objectives:**

Photon-counting computed tomography (PCCT) enables quantitative assessment of tissue iodine distribution. This exploratory study investigated associations between PCCT-derived iodine parameters and clinical, serologic, and histopathologic characteristics of pancreatic ductal adenocarcinoma (PDAC).

**Materials and methods:**

This retrospective single-center study included 27 patients with histologically confirmed PDAC who underwent pretreatment contrast-enhanced PCCT. Iodine concentration was measured independently by two blinded readers in arterial and portal-venous phases using a whole-tumor ROI on the largest axial section and a solid-component ROI. Absolute iodine concentration, tumor-to-aorta normalized iodine ratios, and phase-to-phase Δ iodine were evaluated. CA 19 − 9 was analyzed continuously using Spearman rank correlation; cutoff-based and clinicopathologic subgroup comparisons were considered secondary exploratory analyses and were re-analyzed using two-sided Wilcoxon rank-sum tests. All p-values are nominal.

**Results:**

CA 19 − 9 was available in 24 patients (median 123.5 U/mL; IQR 55.3–1962). Higher CA 19 − 9 was associated with lower portal-venous absolute iodine concentration in the solid tumor component (Spearman ρ=-0.45, 95% CI -0.77 to -0.04; nominal *p* = 0.028) and with the arterial whole-tumor normalized iodine ratio (ρ=-0.58, 95% CI -0.84 to -0.14; nominal *p* = 0.010). At the 100 U/mL cutoff, portal-venous solid-component absolute iodine concentration was higher below the cutoff (1.77 ± 0.57 vs. 1.19 ± 0.50 mg/mL; nominal *p* = 0.017). After two-sided re-analysis, previously reported differences by T category, Ki-67, and tumor-cell count did not reach nominal significance. Interreader reliability was excellent across iodine parameters (ICC range 0.960–0.989).

**Conclusion:**

In this small exploratory cohort, selected absolute and normalized iodine measurements showed nominal inverse associations with CA 19 − 9, whereas associations with tumor stage and histopathologic parameters were not consistent after conservative two-sided re-analysis. PCCT iodine measurements were highly reproducible between readers. These findings are hypothesis-generating and require validation in larger independent cohorts before any incremental clinical value can be inferred.

## Introduction

Pancreatic ductal adenocarcinoma (PDAC) is the most common malignant tumor of the pancreas and remains associated with poor survival despite advances in imaging and oncologic therapy [1–3]. A major clinical challenge is that PDAC is frequently diagnosed at an advanced stage, when curative treatment options are limited. Accordingly, improved early detection and more informative pretreatment characterization remain important research objectives [4].

Contrast-enhanced multidetector CT is central to pancreatic cancer detection, staging, assessment of vascular involvement, and metastatic evaluation [3]. Photon-counting detector CT (PCCT) provides energy-resolved data acquisition with reduced electronic noise, high spatial resolution, and spectral material decomposition capabilities [5–7]. These properties permit quantitative iodine mapping in addition to conventional morphologic assessment.

Iodine concentration reflects contrast-material distribution within tissue and may be influenced by vascularity, extracellular distribution, acquisition timing, and systemic hemodynamics. PDAC commonly demonstrates reduced enhancement in the setting of desmoplastic stroma and altered microvascular architecture [8–11]. Prior dual-energy and spectral CT studies have evaluated iodine-based parameters for tumor conspicuity and quantitative characterization [12–15]. However, PCCT-specific evidence in PDAC remains limited.

Serum carbohydrate antigen 19 − 9 (CA 19 − 9) is widely used in the clinical evaluation of pancreatic cancer and has been associated with tumor burden, stage, and outcome, although levels may also be influenced by benign biliary obstruction and cholestasis [2, 16–21]. Histologic grade, Ki-67 labeling index, and tumor-cell count within a defined high-power field represent complementary pathological characteristics [11, 22, 23]. The aim of the present exploratory study was to evaluate associations between PCCT-derived iodine measurements and CA 19 − 9, TNM category, histologic grade, Ki-67, and tumor-cell count in the pretreatment setting.

## Materials and methods

### Study design and patient population

This retrospective exploratory single-center study evaluated patients with histologically confirmed PDAC who underwent contrast-enhanced PCCT before oncologic treatment between September 2021 and June 2024. Pretreatment imaging was selected to avoid potential effects of systemic therapy on tumor enhancement.

**Fig. 1:**
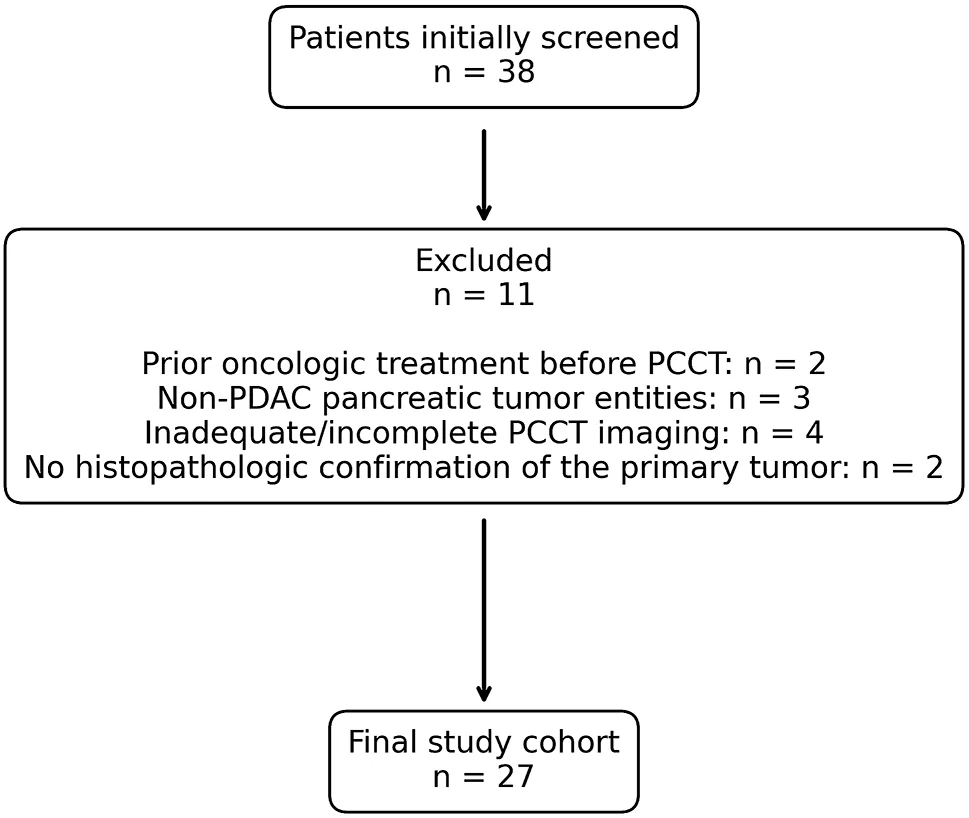
Patient selection flow diagram

A total of 38 patients were initially screened. Eleven were excluded: two because of prior oncologic treatment before PCCT, three because of non-PDAC pancreatic tumor entities, four because of inadequate or incomplete PCCT imaging data, and two because histopathologic confirmation of the primary tumor was unavailable. The final cohort comprised 27 patients (17 men and 10 women). Patient selection is summarized in Fig. 1.

Clinical variables were retrieved retrospectively from the electronic medical record. TNM classification followed the 8th edition of the Union for International Cancer Control (UICC) system. N1 was defined as metastasis in 1–3 regional lymph nodes and N2 as metastasis in ≥ 4 regional lymph nodes. In the source dataset, T category was derived from surgical resection pathology in 16 patients (pT) and from clinical/radiologic staging in 11 patients (cT). N category was derived from surgical resection pathology in 16 patients (pN), from clinical/radiologic staging in 8 patients (cN), and was unavailable in 3 patients. The 16 pathologically staged cases contained pT/pN rather than ypT/ypN designations; however, the source dataset did not systematically retain treatment dates between PCCT and subsequent resection, so treatment intervals could not be independently reconstructed for every patient.

In all 11 patients with missing M category, the source staging record contained pT/pN resection staging without a documented M category. These cases were retained as missing rather than recoded as M0. Histologic grading was extracted from the available pathology documentation using the documented G1–G4 differentiation categories; the retrospective source dataset did not consistently preserve the specimen source used for grading on a patient-by-patient basis. Data availability otherwise differed by variable: grading was available in 24 patients, CA 19 − 9 in 24, and Ki-67 and tumor-cell count in 17 patients each.

### Photon-counting computed tomography

All examinations were performed using a dual-source photon-counting CT scanner (NAEOTOM Alpha, Siemens Healthineers, Erlangen, Germany). Imaging was performed after intravenous administration of iodinated contrast material (Solutrast 300 mg iodine/mL, Bracco Imaging Deutschland GmbH) at approximately 1 mL/kg body weight, followed by a saline flush at 2.5 mL/s. Bolus tracking was performed in the descending thoracic aorta using a threshold of 130 HU. Arterial-phase acquisition was initiated 15 s after reaching the threshold and portal-venous acquisition 60 s after threshold enhancement. Both phases covered the abdomen from the diaphragm to the iliac crest.

Acquisition parameters included 140 kVp, automatic tube-current modulation, detector collimation of 144 × 0.4 mm, pitch 0.8, and rotation time 0.5 s. Axial images were reconstructed at 1-mm thickness. Polyenergetic images were reconstructed using a soft-tissue kernel, while spectral datasets were reconstructed using the Qr36 kernel with Quantum Iterative Reconstruction level Q4. Spectral post-processing was performed with the CT Dual Energy workflow in syngo.via VB80 (Siemens Healthineers). Quantitative iodine concentration was measured directly on vendor-generated iodine maps. Material decomposition used the vendor-specific implementation without additional user-defined material-basis settings. Virtual non-contrast images and calcium-subtraction algorithms were not used for quantitative analysis.

### Iodine concentration analysis

Image analysis was performed independently by two readers with five years and one year of experience in pancreatic imaging, respectively. Both readers were blinded to the clinical, serologic, and histopathologic comparison parameters during iodine measurement. For association analyses, the mean of both reader measurements was used.

**Fig. 2:**
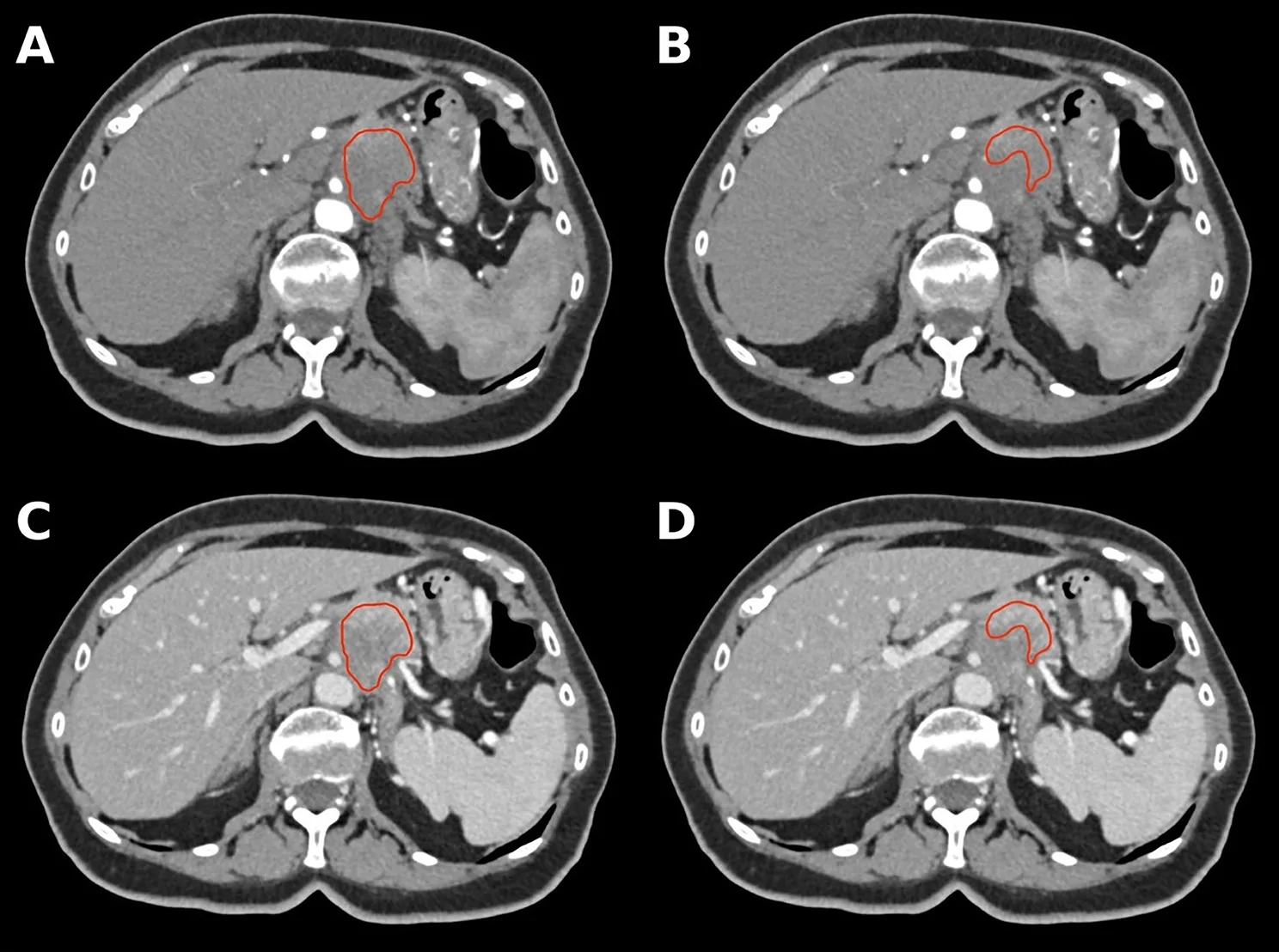
Representative photon-counting CT (PCCT) images illustrating region-of-interest (ROI) placement in a pancreatic head PDAC. **A**–**B**: arterial phase; **C**–**D**: portal-venous phase. **A** and **C** show the whole-tumor ROI on the largest axial section; **B** and **D** show the solid-component ROI, excluding visible central necrotic or cystic areas. Red contours indicate the ROIs used for iodine concentration measurements

For each tumor, the axial section demonstrating the largest cross-sectional tumor extent was selected. A manually drawn whole-tumor ROI on this largest axial section encompassed the visible tumor while avoiding adjacent normal pancreatic parenchyma. A second, smaller ROI was positioned within the peripheral solid tumor component and excluded visible central necrotic or cystic areas. An additional circular ROI was placed within the abdominal aorta on the corresponding axial section. The exact aortic ROI area was not consistently recorded in the retrospective dataset. Representative ROI placement is shown in Fig. 2.

Absolute iodine concentration was recorded in mg/mL in arterial and portal-venous phases. The normalized iodine concentration was calculated as the dimensionless tumor-to-aorta iodine ratio to reduce interindividual variability in systemic contrast distribution. Δ absolute iodine concentration was defined as portal-venous minus arterial absolute iodine concentration and was calculated only when both phases were available. The analogous Δ normalized iodine ratio was calculated for normalized measurements. Absolute and normalized parameters were interpreted separately.

### Histopathological evaluation

Histopathologic analyses of Ki-67 and tumor-cell count were based on pretreatment biopsies of the primary pancreatic tumor; surgical resection specimens and metastatic biopsies were not used for these analyses. Tissue sampling was performed under sterile conditions using 18-gauge coaxial biopsy needles, with two to three cylindrical tissue cores obtained during routine clinical procedures. Specimens were formalin-fixed, paraffin-embedded, and cut into 2-µm sections. Hematoxylin-eosin staining and immunohistochemical staining were performed.

Ki-67 expression was retrospectively assessed using a monoclonal mouse antibody (clone MIB1; dilution 1:300), with CC1 mild antigen retrieval, incubation at 36 °C for 40 min, and detection using the ultraView DAB Detection Kit (Roche Diagnostics, Basel, Switzerland). Histopathologic evaluation was performed by a board-certified pathologist with 20 years of experience. Ki-67 and tumor-cell count were assessed in one high-power field (HPF; ×400; 0.23 mm²) per tumor. The Ki-67 labeling index was defined as the percentage of Ki-67-positive tumor cells relative to the total tumor-cell count within the analyzed field. Tumor-cell count denotes the absolute number of tumor cells within this 0.23-mm² field and not a proportional measure of tumor cellularity. Microscopic images were digitally documented using a Nikon ECLIPSE Ni-E microscope and Nikon DS-Ri2 camera.

The archived study documentation did not specify a standardized rule for selection of the single HPF (for example, hotspot versus representative-field selection) or whether tumor-cell enumeration itself was performed manually or with software assistance; digital image acquisition documented the field but does not establish the counting modality. Formal blinding of the pathologic assessment to imaging measurements was not prospectively documented. Exact patient-level intervals between PCCT, biopsy, and CA 19 − 9 sampling could not be reconstructed because complete specimen- and laboratory-level dates were not retained in the structured source dataset.

### CA 19 − 9 and clinicopathologic variables

Serum CA 19 − 9 values were obtained from routine pretreatment laboratory testing. Because CA 19 − 9 was markedly right-skewed, continuous-variable analyses were emphasized in the revised analysis. The thresholds of 100, 500, and 1000 U/mL were part of the original analytical framework; cutoff-based comparisons were retained as secondary exploratory analyses. The 100 U/mL threshold has prior prognostic precedent in PDAC [19], whereas the higher thresholds were exploratory. Continuous tumor diameter and systematic patient-level information on tumor location, bilirubin/cholestasis, and biliary drainage or stenting were not included in the structured source dataset and could not be reconstructed uniformly from the archived study material.

### Statistical analysis

The original statistical analyses were performed using JMP Statistical Discovery Software (version 18.0.2; SAS Institute, Cary, NC, USA). For the revision, all reported inferential statistics were independently recalculated from the source dataset in Python 3.13.5 using SciPy 1.17.0. Distributional assumptions were evaluated using the Shapiro-Wilk test. Review of the original analytical workflow showed that one-sided Wilcoxon tests had been used for directional two-group hypotheses involving iodine parameters, Ki-67, and tumor-cell count, including the original CA 19 − 9 cutoff and clinicopathologic subgroup comparisons. For the revised analysis, all such group comparisons were recalculated consistently using two-sided Wilcoxon rank-sum (Mann-Whitney U) tests.

Associations between continuous CA 19 − 9 and iodine parameters were assessed using two-sided Spearman rank correlation. Nominal Spearman p-values were the standard two-sided values returned by scipy.stats.spearmanr. 95% confidence intervals for Spearman coefficients were obtained by nonparametric percentile bootstrap resampling of paired observations (10,000 resamples). Given the small sample size and the borderline arterial whole-tumor result, two-sided Monte Carlo permutation tests were additionally performed as sensitivity analyses for the three CA 19 − 9–iodine correlations with nominal *p* < 0.05 using 1,000,000 random permutations; the permutation p-value was calculated using the absolute Spearman coefficient and a + 1 correction.

No adjustment for multiple comparisons was applied because the study was exploratory and no single confirmatory primary endpoint had been prospectively prespecified. Accordingly, all p-values are reported as nominal and should be interpreted as hypothesis-generating. A p-value < 0.05 was not considered confirmatory evidence.

Interreader reliability was assessed using a two-way random-effects intraclass correlation coefficient for absolute agreement of single measurements (ICC[A,1], equivalent to ICC [1, 2]) using paired patient-level reader measurements. 95% confidence intervals were estimated by nonparametric percentile bootstrap resampling (10,000 resamples). Bland-Altman analysis was additionally performed for the portal-venous solid-component absolute iodine measurement; differences were defined as Reader 2 minus Reader 1.

## Results

### Patient characteristics

The final cohort consisted of 27 patients with PDAC (17 men, 10 women; mean age 70.9 ± 7.0 years). CA 19 − 9 was available in 24 patients and was markedly right-skewed, with a median of 123.5 U/mL (IQR 55.3–1962; range 14 to > 10,000 U/mL). Detailed cohort characteristics and variable availability are summarized in Table 1.

**Table 1:** Patient and tumor characteristics

Characteristic	Value
Patients	27
Age, years	70.9 ± 7.0
Male / female	17 / 10
T category	T2: 11; T3: 9; T4: 7
N category	N0: 8; N1: 10; N2: 6; missing: 3
M category	M0: 11; M1: 5; missing: 11
Histologic grade	G2: 9; G3: 14; G4: 1; missing: 3
CA 19 − 9 available	24/27; median 123.5 U/mL (IQR 55.3–1962)
Ki-67 available	17/27; mean 38.6 ± 16.8%
Tumor-cell count available	17/27; mean 124.9 ± 35.6 cells/0.23-mm² HPF

### Continuous association between CA 19 − 9 and iodine parameters

**Fig. 3:**
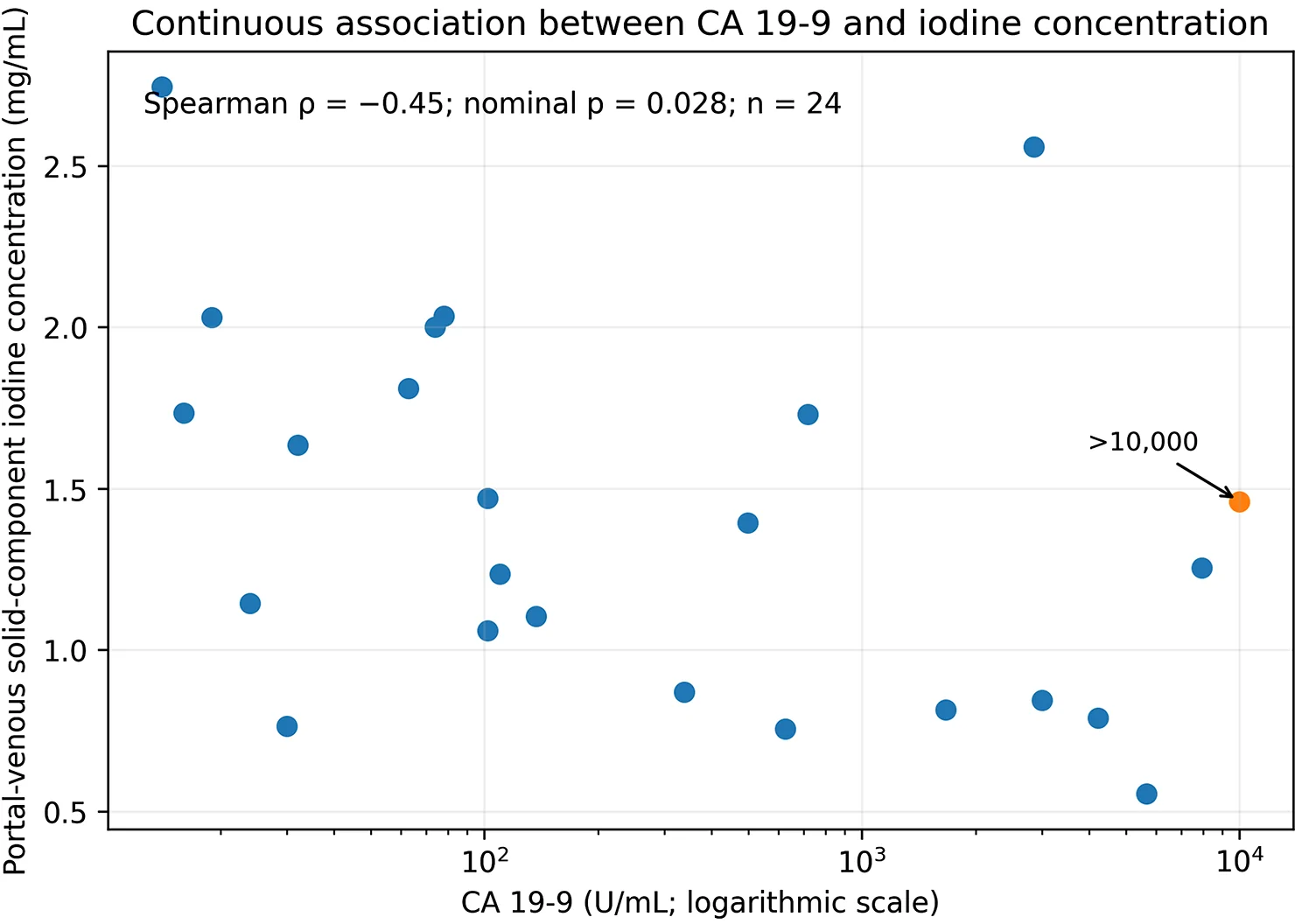
Continuous relationship between serum CA 19 − 9 and portal-venous absolute iodine concentration in the solid-component ROI. CA 19 − 9 is shown on a logarithmic x-axis. One right-censored laboratory value reported as > 10,000 U/mL is displayed at 10,000 U/mL solely for visualization; this does not affect its rank in the Spearman analysis. Statistical inference is based on Spearman rank correlation (ρ = −0.45, nominal *p* = 0.028; *n* = 24)

In continuous analyses, higher CA 19 − 9 was associated with lower portal-venous absolute iodine concentration in the solid-component ROI (*n* = 24; Spearman ρ=-0.45, 95% CI -0.77 to -0.04; nominal *p* = 0.028; Fig. 3). An inverse association was also observed for the arterial whole-tumor normalized iodine ratio (*n* = 19; ρ=-0.58, 95% CI -0.84 to -0.14; nominal *p* = 0.010). The arterial whole-tumor absolute iodine concentration showed a borderline inverse correlation (*n* = 19; ρ=-0.46, bootstrap 95% CI -0.81 to 0.02; nominal *p* = 0.047). Two-sided permutation sensitivity analyses yielded similar p-values for these three associations (*p* = 0.029, *p* = 0.011, and *p* = 0.049, respectively). Because the bootstrap confidence interval for the arterial whole-tumor absolute iodine correlation included zero, this finding was interpreted as borderline and with particular caution. The remaining continuous correlations were not nominally significant (Table 2).

**Table 2:** Continuous Spearman associations between CA 19 − 9 and PCCT-derived iodine parameters

Parameter	*n*	Spearman ρ	95% CI	Nominal *p*
Whole-tumor PV absolute IC (mg/mL)	24	-0.33	-0.70 to 0.10	0.111
Whole-tumor arterial absolute IC (mg/mL)	19	-0.46	-0.81 to 0.02	0.047
Whole-tumor Δ absolute IC (mg/mL)	19	-0.19	-0.63 to 0.35	0.446
Solid-component PV absolute IC (mg/mL)	24	-0.45	-0.77 to -0.04	0.028
Solid-component arterial absolute IC (mg/mL)	19	-0.25	-0.70 to 0.31	0.304
Solid-component Δ absolute IC (mg/mL)	19	-0.32	-0.67 to 0.14	0.179
Whole-tumor PV normalized iodine ratio	24	-0.21	-0.58 to 0.22	0.320
Whole-tumor arterial normalized iodine ratio	19	-0.58	-0.84 to -0.14	0.010
Whole-tumor Δ normalized iodine ratio	19	-0.21	-0.63 to 0.30	0.399
Solid-component PV normalized iodine ratio	24	-0.30	-0.66 to 0.16	0.157
Solid-component arterial normalized iodine ratio	19	-0.29	-0.71 to 0.26	0.226
Solid-component Δ normalized iodine ratio	19	-0.23	-0.67 to 0.29	0.351

Confidence intervals are percentile bootstrap 95% CIs based on 10,000 resamples. All p-values are nominal

### Secondary CA 19 − 9 cutoff analyses

At the 100 U/mL cutoff, portal-venous absolute iodine concentration in the solid-component ROI was higher in patients with CA 19 − 9 < 100 U/mL than in those with CA 19 − 9 ≥ 100 U/mL (1.77 ± 0.57 vs. 1.19 ± 0.50 mg/mL; nominal *p* = 0.017). Whole-tumor portal-venous, whole-tumor arterial, and solid-component Δ absolute iodine differences that had been reported as significant in the submitted manuscript no longer reached nominal significance after two-sided re-analysis (*p* = 0.056, *p* = 0.098, and *p* = 0.063, respectively).

At the 500 and 1000 U/mL cutoffs, none of the absolute iodine comparisons reached nominal *p* < 0.05 after two-sided re-analysis (Table 3). The previously described reversal at the 1000 U/mL threshold was a transcription error: arterial whole-tumor iodine remained higher below the cutoff (0.49 ± 0.25 vs. 0.29 ± 0.18 mg/mL; nominal *p* = 0.147). Normalized cutoff analyses were not uniformly concordant with absolute iodine findings and are provided in Supplementary Table S1.

**Table 3:** Secondary exploratory CA 19 − 9 cutoff analyses for absolute iodine concentration

Parameter	Lower CA 19 − 9 group	Higher CA 19 − 9 group	Nominal *p*
CA 19 − 9 cutoff 100 U/mL			
Whole-tumor PV	1.16 ± 0.64 (*n* = 9)	0.72 ± 0.43 (*n* = 15)	0.056
Whole-tumor arterial	0.57 ± 0.27 (*n* = 8)	0.36 ± 0.20 (*n* = 11)	0.098
Whole-tumor Δ	0.66 ± 0.48 (*n* = 8)	0.43 ± 0.40 (*n* = 11)	0.177
Solid-component PV	1.77 ± 0.57 (*n* = 9)	1.19 ± 0.50 (*n* = 15)	0.017
Solid-component arterial	0.90 ± 0.49 (*n* = 8)	0.83 ± 0.38 (*n* = 11)	0.840
Solid-component Δ	0.87 ± 0.36 (*n* = 8)	0.44 ± 0.53 (*n* = 11)	0.063
CA 19 − 9 cutoff 500 U/mL			
Whole-tumor PV	0.95 ± 0.57 (*n* = 15)	0.78 ± 0.53 (*n* = 9)	0.371
Whole-tumor arterial	0.50 ± 0.25 (*n* = 13)	0.32 ± 0.22 (*n* = 6)	0.174
Whole-tumor Δ	0.50 ± 0.42 (*n* = 13)	0.58 ± 0.50 (*n* = 6)	0.765
Solid-component PV	1.54 ± 0.54 (*n* = 15)	1.20 ± 0.64 (*n* = 9)	0.095
Solid-component arterial	0.93 ± 0.43 (*n* = 13)	0.71 ± 0.39 (*n* = 6)	0.323
Solid-component Δ	0.63 ± 0.53 (*n* = 13)	0.60 ± 0.49 (*n* = 6)	0.456
CA 19 − 9 cutoff 1000 U/mL			
Whole-tumor PV	0.94 ± 0.54 (*n* = 17)	0.75 ± 0.58 (*n* = 7)	0.349
Whole-tumor arterial	0.49 ± 0.25 (*n* = 15)	0.29 ± 0.18 (*n* = 4)	0.147
Whole-tumor Δ	0.49 ± 0.39 (*n* = 15)	0.63 ± 0.63 (*n* = 4)	0.961
Solid-component PV	1.50 ± 0.54 (*n* = 17)	1.18 ± 0.68 (*n* = 7)	0.166
Solid-component arterial	0.91 ± 0.41 (*n* = 15)	0.68 ± 0.49 (*n* = 4)	0.411
Solid-component Δ	0.61 ± 0.52 (*n* = 15)	0.66 ± 0.52 (*n* = 4)	0.653

Values are mean ± SD; p-values are from two-sided Wilcoxon rank-sum tests and are nominal

### Histopathologic parameters and tumor stage

Tumor-cell count was 107.5 ± 15.5 cells in patients with CA 19 − 9 < 100 U/mL and 152.7 ± 41.6 cells in patients with CA 19 − 9 ≥ 100 U/mL; the difference did not reach nominal significance after two-sided re-analysis (*p* = 0.070). Continuous CA 19 − 9 was not associated with tumor-cell count (*n* = 14; ρ = 0.21; *p* = 0.463).

Ki-67 values were stored as proportions in the source dataset and are reported here as percentages. Mean Ki-67 was 45.8 ± 14.6% in T1–T2 tumors and 33.6 ± 17.0% in T3–T4 tumors; the difference did not reach nominal significance after two-sided re-analysis (*p* = 0.088). Continuous CA 19 − 9 was not associated with Ki-67 (*n* = 14; ρ=-0.14; *p* = 0.637). Exploratory continuous analyses did not identify nominally significant correlations between any evaluated absolute or normalized iodine parameter and either Ki-67 labeling index or tumor-cell count. Detailed sample sizes, Spearman coefficients, bootstrap 95% confidence intervals, and nominal p-values are provided in Supplementary Table S4.

Portal-venous whole-tumor absolute iodine concentration was numerically higher in T1–T2 than in T3–T4 tumors (1.10 ± 0.62 vs. 0.74 ± 0.40 mg/mL), but the difference did not reach nominal significance after two-sided re-analysis (*p* = 0.098). No nominally significant differences were identified for the other absolute iodine parameters by T category (Table 4), or by nodal status, metastatic status, or histologic grade (Supplementary Table S2).

**Table 4:** Exploratory comparisons by T category and selected histopathologic variables after two-sided re-analysis

Parameter	Group A	Group B	Nominal *p*
T category: T1–T2 vs. T3–T4			
Whole-tumor PV	1.10 ± 0.62 (*n* = 11)	0.74 ± 0.40 (*n* = 16)	0.098
Whole-tumor arterial	0.56 ± 0.32 (*n* = 9)	0.40 ± 0.18 (*n* = 13)	0.242
Whole-tumor Δ	0.62 ± 0.53 (*n* = 9)	0.41 ± 0.30 (*n* = 13)	0.385
Solid-component PV	1.54 ± 0.66 (*n* = 11)	1.32 ± 0.49 (*n* = 16)	0.570
Solid-component arterial	1.00 ± 0.48 (*n* = 9)	0.78 ± 0.33 (*n* = 13)	0.182
Solid-component Δ	0.67 ± 0.43 (*n* = 9)	0.55 ± 0.51 (*n* = 13)	0.404
Ki-67 labeling index (%)	45.8 ± 14.6 (*n* = 7)	33.6 ± 17.0 (*n* = 10)	0.088
CA 19 − 9: <100 vs. ≥ 100 U/mL			
Tumor-cell count (cells/0.23-mm² HPF)	107.5 ± 15.5 (*n* = 8)	152.7 ± 41.6 (*n* = 6)	0.070

Values are mean ± SD; group order is specified in the shaded rows; p-values are nominal

### Interreader agreement

Interreader reliability was excellent across all absolute, normalized, and Δ iodine parameters, with ICC values ranging from 0.960 to 0.989 (Supplementary Table S3). For the portal-venous solid-component absolute iodine measurement, ICC was 0.974 (95% CI 0.936–0.991). Bland-Altman analysis showed a mean Reader 2–Reader 1 bias of 0.002 mg/mL, with 95% limits of agreement from − 0.259 to 0.263 mg/mL (Supplementary Figure S1).

## Discussion

This exploratory study found nominal inverse correlations between selected PCCT-derived iodine measurements and continuous CA 19 − 9, most clearly for portal-venous absolute iodine concentration in the solid tumor component and the arterial whole-tumor normalized iodine ratio. However, these associations were not uniform across phases, ROI strategies, normalization, Δ measurements, or cutoff analyses. After re-analysis using two-sided nonparametric tests, previously reported borderline findings for T category, Ki-67, and tumor-cell count were no longer nominally significant. These results therefore warrant cautious, hypothesis-generating interpretation rather than biomarker or mechanistic claims.

The continuous CA 19 − 9 analysis reduces the information loss inherent in repeated dichotomization. The inverse association observed for portal-venous solid-component iodine may indicate that tumors associated with higher CA 19 − 9 show different contrast-material accumulation; however, iodine concentration is not a direct measurement of perfusion or microvascular density. It is influenced by contrast delivery, extracellular distribution, acquisition timing, vascular anatomy, and systemic hemodynamics [8–10, 12]. In addition, CA 19 − 9 itself can be influenced by biliary obstruction and cholestasis [19]. Bilirubin, biliary drainage/stenting, and tumor location were not consistently available in the structured retrospective dataset and could not be incorporated as covariates. Reconstructing these variables would have required a new post hoc PACS/EHR re-abstraction with retrospectively defined imaging and laboratory time points, which was not undertaken.

The submitted manuscript interpreted higher iodine concentration in T1–T2 tumors as potentially reflecting greater vascularization. This interpretation is not supported after conservative two-sided re-analysis, in which the T-category comparison no longer reached nominal significance. Moreover, UICC T category in PDAC is closely related to tumor size and local vascular involvement. Continuous tumor diameter was not consistently available in the structured dataset, and the present study cannot separate effects of size, composition, location, vascular encasement, or treatment pathway. Accordingly, no independent stage-related effect is claimed.

Similarly, the absence of consistent iodine associations with Ki-67, tumor-cell count, and grade argues against describing iodine concentration as a direct surrogate of overall tumor biology. The Ki-67 and tumor-cell-count analyses were derived from limited primary-tumor biopsy material and may not capture intratumoral heterogeneity. These analyses were available in only 17 patients, further limiting precision. These negative findings should not be interpreted as evidence that no association exists, because the cohort is underpowered for modest effects.

The excellent interreader ICCs support the technical reproducibility of the measurement approach. Nevertheless, reproducibility does not establish incremental clinical utility. The present study was not designed to determine whether iodine measurements improve diagnosis, staging, prognosis, treatment selection, or response prediction beyond established clinical variables such as tumor size and CA 19 − 9. Multivariable analysis was not performed because *n* = 27 would create a substantial risk of overfitting. Likewise, no head-to-head comparison with dual-energy CT was available; superiority of PCCT over DECT therefore cannot be inferred from this cohort. Related PCCT work in esophageal cancer has also demonstrated the feasibility and reproducibility of normalized iodine quantification and reported an association with histopathologic treatment response [24]; however, findings from another tumor entity cannot be extrapolated to PDAC.

Normalization to aortic iodine concentration was intended to reduce interindividual variation in systemic contrast distribution. Importantly, normalized results were not uniformly concordant with absolute iodine findings. This suggests that absolute and normalized iodine parameters should not be treated as interchangeable. Future studies should prospectively standardize contrast timing, incorporate volumetric or three-dimensional tumor segmentation, and evaluate absolute and normalized measures within appropriately powered multivariable models.

The present study used a standardized two-dimensional ROI on the largest axial tumor section rather than whole-volume segmentation. This approach is practical and reproducible but may undersample the marked spatial heterogeneity of PDAC. Future work could combine volumetric PCCT iodine quantification with complementary MRI or molecular imaging parameters to assess whether multimodality information provides incremental value beyond morphology and serum biomarkers [25].

Several limitations should therefore be emphasized. The study was retrospective, single-center, and small, with incomplete availability of some clinical and histopathologic variables and no multiplicity adjustment. All p-values are nominal and several analyses were secondary exploratory comparisons. Arterial-phase data were unavailable in five patients. Continuous tumor diameter and systematic information on bilirubin, cholestasis, biliary drainage/stenting, and tumor location were unavailable. Exact treatment intervals between PCCT and subsequent resection pathology were not systematically retained. For the Ki-67 and tumor-cell-count analyses, the retrospective documentation did not preserve a standardized single-HPF selection rule or the counting modality (manual versus software-assisted), and exact PCCT-to-biopsy/CA 19 − 9 intervals were not recoverable. The specimen source used for histologic grading could not be distinguished reliably for every patient. Formal blinding of the pathologist to imaging measurements was not prospectively documented. Manual single-slice ROI placement may not capture whole-tumor heterogeneity. No multivariable model or direct DECT comparator was feasible, and only baseline imaging was analyzed. These limitations preclude conclusions regarding independent prognostic value or clinical decision-making.

## Conclusion

In this small exploratory cohort, selected PCCT-derived absolute and normalized iodine measurements showed nominal inverse associations with continuous CA 19 − 9, while associations with T category and histopathologic parameters were not consistently supported after two-sided re-analysis. Interreader reproducibility was excellent. The findings should be considered hypothesis-generating and do not establish iodine concentration as an independent biomarker, a direct perfusion surrogate, or a clinically superior alternative to established imaging and laboratory parameters. Larger prospective studies with standardized volumetric measurements, relevant clinical confounders, and multivariable validation are required.

## Supplementary Information

Below is the link to the electronic supplementary material.

Supplementary Material 1

Supplementary Material 2

Supplementary Material 3

## Data Availability

The datasets generated and/or analyzed during the current study are available from the corresponding author on reasonable request.

## Abbreviations

PCCT: Photon-counting computed tomography
PDAC: Pancreatic ductal adenocarcinoma
CA 19 − 9: Carbohydrate antigen 19 − 9
ROI: Region of interest
ICC: Intraclass correlation coefficient
HPF: High-power field
UICC: Union for International Cancer Control
DECT: Dual-energy computed tomography
