# Supplementary material for: Photon-counting computed tomography in pancreatic ductal adenocarcinoma: correlation of iodine concentration with histopathological and clinical parameters
Source: BMC Med Imaging. 2026 Nov 4;26:583. doi: 10.1186/s12880-026-02980-2 (PMC13737619; doi:10.1186/s12880-026-02980-2)
Supplement: Supplementary file 2 [file 12880_2026_2980_MOESM2_ESM.docx]

**Supplementary Material**

Photon-counting computed tomography in pancreatic ductal adenocarcinoma: correlation of iodine concentration with histopathological and clinical parameters

**Supplementary Table S1.** Secondary exploratory CA 19-9 cutoff analyses for normalized tumor-to-aorta iodine ratios. Values are mean ± SD. Ratios are dimensionless; p-values are two-sided Wilcoxon rank-sum tests and are nominal.

| **Parameter** | **Lower CA 19-9 group** | **Higher CA 19-9 group** | **Nominal p** |
| --- | --- | --- | --- |
| CA 19-9 cutoff 100 U/mL |  |  |  |
| Whole-tumor PV ratio | 0.30 ± 0.18 (n=9) | 0.21 ± 0.10 (n=15) | 0.404 |
| Whole-tumor arterial ratio | 0.04 ± 0.02 (n=8) | 0.03 ± 0.01 (n=11) | 0.152 |
| Whole-tumor Δ normalized ratio | 0.27 ± 0.18 (n=8) | 0.20 ± 0.09 (n=11) | 0.600 |
| Solid-component PV ratio | 0.46 ± 0.19 (n=9) | 0.35 ± 0.10 (n=15) | 0.340 |
| Solid-component arterial ratio | 0.07 ± 0.03 (n=8) | 0.07 ± 0.03 (n=11) | 1.000 |
| Solid-component Δ normalized ratio | 0.39 ± 0.19 (n=8) | 0.30 ± 0.09 (n=11) | 0.395 |
| CA 19-9 cutoff 500 U/mL |  |  |  |
| Whole-tumor PV ratio | 0.27 ± 0.16 (n=15) | 0.21 ± 0.10 (n=9) | 0.474 |
| Whole-tumor arterial ratio | 0.04 ± 0.02 (n=13) | 0.02 ± 0.01 (n=6) | 0.029 |
| Whole-tumor Δ normalized ratio | 0.25 ± 0.15 (n=13) | 0.21 ± 0.08 (n=6) | 0.639 |
| Solid-component PV ratio | 0.44 ± 0.15 (n=15) | 0.32 ± 0.11 (n=9) | 0.107 |
| Solid-component arterial ratio | 0.08 ± 0.03 (n=13) | 0.05 ± 0.02 (n=6) | 0.152 |
| Solid-component Δ normalized ratio | 0.37 ± 0.15 (n=13) | 0.29 ± 0.11 (n=6) | 0.416 |
| CA 19-9 cutoff 1000 U/mL |  |  |  |
| Whole-tumor PV ratio | 0.27 ± 0.15 (n=17) | 0.19 ± 0.11 (n=7) | 0.349 |
| Whole-tumor arterial ratio | 0.04 ± 0.02 (n=15) | 0.02 ± 0.01 (n=4) | 0.062 |
| Whole-tumor Δ normalized ratio | 0.24 ± 0.14 (n=15) | 0.20 ± 0.09 (n=4) | 0.596 |
| Solid-component PV ratio | 0.43 ± 0.14 (n=17) | 0.31 ± 0.10 (n=7) | 0.087 |
| Solid-component arterial ratio | 0.07 ± 0.03 (n=15) | 0.05 ± 0.03 (n=4) | 0.221 |
| Solid-component Δ normalized ratio | 0.36 ± 0.15 (n=15) | 0.27 ± 0.11 (n=4) | 0.411 |

**Supplementary Table S2.** Exploratory absolute and normalized iodine comparisons by nodal status, metastatic status, and histologic grade. Values are mean ± SD. All p-values are nominal two-sided Wilcoxon rank-sum tests.

| **Grouping** | **Parameter** | **Group 0** | **Group 1** | **Nominal p** |
| --- | --- | --- | --- | --- |
| N0 vs N1/N2 | Whole-tumor PV absolute IC (mg/mL) | 0.97 ± 0.52 (n=8) | 0.90 ± 0.57 (n=16) | 0.742 |
| N0 vs N1/N2 | Whole-tumor arterial absolute IC (mg/mL) | 0.51 ± 0.25 (n=6) | 0.50 ± 0.26 (n=13) | 0.965 |
| N0 vs N1/N2 | Whole-tumor Δ absolute IC (mg/mL) | 0.61 ± 0.51 (n=6) | 0.47 ± 0.41 (n=13) | 0.579 |
| N0 vs N1/N2 | Solid-component PV absolute IC (mg/mL) | 1.63 ± 0.58 (n=8) | 1.34 ± 0.58 (n=16) | 0.192 |
| N0 vs N1/N2 | Solid-component arterial absolute IC (mg/mL) | 1.03 ± 0.34 (n=6) | 0.86 ± 0.43 (n=13) | 0.323 |
| N0 vs N1/N2 | Solid-component Δ absolute IC (mg/mL) | 0.65 ± 0.76 (n=6) | 0.58 ± 0.38 (n=13) | 0.861 |
| N0 vs N1/N2 | Whole-tumor PV normalized iodine ratio | 0.24 ± 0.09 (n=8) | 0.26 ± 0.16 (n=16) | 0.976 |
| N0 vs N1/N2 | Whole-tumor arterial normalized iodine ratio | 0.04 ± 0.02 (n=6) | 0.04 ± 0.02 (n=13) | 0.579 |
| N0 vs N1/N2 | Whole-tumor Δ normalized iodine ratio | 0.24 ± 0.07 (n=6) | 0.23 ± 0.15 (n=13) | 0.701 |
| N0 vs N1/N2 | Solid-component PV normalized iodine ratio | 0.40 ± 0.08 (n=8) | 0.39 ± 0.16 (n=16) | 0.610 |
| N0 vs N1/N2 | Solid-component arterial normalized iodine ratio | 0.08 ± 0.03 (n=6) | 0.07 ± 0.03 (n=13) | 0.701 |
| N0 vs N1/N2 | Solid-component Δ normalized iodine ratio | 0.33 ± 0.09 (n=6) | 0.34 ± 0.16 (n=13) | 0.898 |
| M0 vs M1 | Whole-tumor PV absolute IC (mg/mL) | 0.77 ± 0.42 (n=11) | 0.86 ± 0.71 (n=5) | 1.000 |
| M0 vs M1 | Whole-tumor arterial absolute IC (mg/mL) | 0.33 ± 0.19 (n=8) | 0.38 ± 0.19 (n=4) | 0.683 |
| M0 vs M1 | Whole-tumor Δ absolute IC (mg/mL) | 0.49 ± 0.35 (n=8) | 0.66 ± 0.63 (n=4) | 0.933 |
| M0 vs M1 | Solid-component PV absolute IC (mg/mL) | 1.28 ± 0.49 (n=11) | 1.44 ± 0.80 (n=5) | 0.827 |
| M0 vs M1 | Solid-component arterial absolute IC (mg/mL) | 0.61 ± 0.24 (n=8) | 0.83 ± 0.43 (n=4) | 0.283 |
| M0 vs M1 | Solid-component Δ absolute IC (mg/mL) | 0.75 ± 0.46 (n=8) | 0.77 ± 0.51 (n=4) | 0.933 |
| M0 vs M1 | Whole-tumor PV normalized iodine ratio | 0.24 ± 0.15 (n=11) | 0.19 ± 0.11 (n=5) | 0.743 |
| M0 vs M1 | Whole-tumor arterial normalized iodine ratio | 0.03 ± 0.01 (n=8) | 0.03 ± 0.01 (n=4) | 0.933 |
| M0 vs M1 | Whole-tumor Δ normalized iodine ratio | 0.22 ± 0.16 (n=8) | 0.19 ± 0.09 (n=4) | 0.933 |
| M0 vs M1 | Solid-component PV normalized iodine ratio | 0.39 ± 0.17 (n=11) | 0.32 ± 0.12 (n=5) | 0.510 |
| M0 vs M1 | Solid-component arterial normalized iodine ratio | 0.05 ± 0.02 (n=8) | 0.06 ± 0.03 (n=4) | 0.461 |
| M0 vs M1 | Solid-component Δ normalized iodine ratio | 0.36 ± 0.19 (n=8) | 0.29 ± 0.10 (n=4) | 0.570 |
| G1/G2 vs G3/G4 | Whole-tumor PV absolute IC (mg/mL) | 0.80 ± 0.36 (n=9) | 0.97 ± 0.63 (n=15) | 0.551 |
| G1/G2 vs G3/G4 | Whole-tumor arterial absolute IC (mg/mL) | 0.42 ± 0.17 (n=7) | 0.52 ± 0.30 (n=12) | 0.554 |
| G1/G2 vs G3/G4 | Whole-tumor Δ absolute IC (mg/mL) | 0.46 ± 0.34 (n=7) | 0.54 ± 0.49 (n=12) | 0.773 |
| G1/G2 vs G3/G4 | Solid-component PV absolute IC (mg/mL) | 1.36 ± 0.45 (n=9) | 1.42 ± 0.68 (n=15) | 1.000 |
| G1/G2 vs G3/G4 | Solid-component arterial absolute IC (mg/mL) | 0.88 ± 0.28 (n=7) | 0.89 ± 0.50 (n=12) | 1.000 |
| G1/G2 vs G3/G4 | Solid-component Δ absolute IC (mg/mL) | 0.54 ± 0.60 (n=7) | 0.60 ± 0.46 (n=12) | 0.352 |
| G1/G2 vs G3/G4 | Whole-tumor PV normalized iodine ratio | 0.25 ± 0.14 (n=9) | 0.25 ± 0.14 (n=15) | 0.721 |
| G1/G2 vs G3/G4 | Whole-tumor arterial normalized iodine ratio | 0.03 ± 0.01 (n=7) | 0.04 ± 0.02 (n=12) | 0.299 |
| G1/G2 vs G3/G4 | Whole-tumor Δ normalized iodine ratio | 0.25 ± 0.14 (n=7) | 0.23 ± 0.13 (n=12) | 0.837 |
| G1/G2 vs G3/G4 | Solid-component PV normalized iodine ratio | 0.43 ± 0.17 (n=9) | 0.36 ± 0.12 (n=15) | 0.512 |
| G1/G2 vs G3/G4 | Solid-component arterial normalized iodine ratio | 0.07 ± 0.02 (n=7) | 0.07 ± 0.04 (n=12) | 1.000 |
| G1/G2 vs G3/G4 | Solid-component Δ normalized iodine ratio | 0.39 ± 0.19 (n=7) | 0.30 ± 0.10 (n=12) | 0.384 |

**Supplementary Table S3.** Interreader reliability for PCCT-derived iodine measurements. ICC = two-way random-effects, absolute-agreement, single-measurement ICC(A,1). Confidence intervals are bootstrap 95% CIs.

| **Measurement** | **n** | **ICC** | **95% CI** | **Mean bias (R2−R1)** | **95% limits of agreement** |
| --- | --- | --- | --- | --- | --- |
| Whole-tumor PV absolute | 27 | 0.988 | 0.970–0.996 | 0.016 mg/mL | -0.145 to 0.177 mg/mL |
| Whole-tumor arterial absolute | 22 | 0.977 | 0.932–0.993 | -0.005 mg/mL | -0.114 to 0.103 mg/mL |
| Solid-component PV absolute | 27 | 0.974 | 0.936–0.991 | 0.002 mg/mL | -0.259 to 0.263 mg/mL |
| Solid-component arterial absolute | 22 | 0.970 | 0.929–0.988 | 0.037 mg/mL | -0.147 to 0.221 mg/mL |
| Whole-tumor PV normalized | 27 | 0.989 | 0.964–0.996 | 0.005 | -0.034 to 0.044 |
| Whole-tumor arterial normalized | 22 | 0.970 | 0.894–0.993 | -0.001 | -0.010 to 0.009 |
| Solid-component PV normalized | 27 | 0.966 | 0.885–0.989 | 0.003 | -0.068 to 0.074 |
| Solid-component arterial normalized | 22 | 0.960 | 0.889–0.988 | 0.003 | -0.014 to 0.021 |
| Whole-tumor Δ absolute | 22 | 0.976 | 0.921–0.990 | 0.029 mg/mL | -0.142 to 0.200 mg/mL |
| Solid-component Δ absolute | 22 | 0.961 | 0.876–0.989 | -0.030 mg/mL | -0.289 to 0.230 mg/mL |
| Whole-tumor Δ normalized | 22 | 0.987 | 0.946–0.996 | 0.008 | -0.030 to 0.046 |
| Solid-component Δ normalized | 22 | 0.970 | 0.856–0.992 | 0.001 | -0.065 to 0.066 |

**Supplementary Table S4. Continuous Spearman associations of PCCT-derived iodine parameters with Ki-67 labeling index and tumor-cell count. Confidence intervals are nonparametric percentile bootstrap 95% CIs based on 10,000 resamples. All p-values are nominal and two-sided.**

**A. Ki-67 labeling index**

| **Parameter** | **n** | **Spearman ρ** | **95% CI** | **Nominal p** |
| --- | --- | --- | --- | --- |
| Whole-tumor PV absolute | 17 | 0.23 | -0.39 to 0.76 | 0.379 |
| Whole-tumor arterial absolute | 15 | -0.06 | -0.67 to 0.58 | 0.830 |
| Whole-tumor Δ absolute | 15 | 0.21 | -0.43 to 0.77 | 0.459 |
| Solid-component PV absolute | 17 | 0.16 | -0.44 to 0.69 | 0.548 |
| Solid-component arterial absolute | 15 | -0.05 | -0.63 to 0.58 | 0.860 |
| Solid-component Δ absolute | 15 | 0.28 | -0.25 to 0.68 | 0.305 |
| Whole-tumor PV normalized | 17 | 0.32 | -0.26 to 0.78 | 0.209 |
| Whole-tumor arterial normalized | 15 | 0.05 | -0.55 to 0.60 | 0.850 |
| Whole-tumor Δ normalized | 15 | 0.31 | -0.32 to 0.83 | 0.254 |
| Solid-component PV normalized | 17 | 0.16 | -0.44 to 0.69 | 0.535 |
| Solid-component arterial normalized | 15 | -0.03 | -0.56 to 0.52 | 0.909 |
| Solid-component Δ normalized | 15 | 0.29 | -0.35 to 0.83 | 0.302 |

**B. Tumor-cell count (absolute cells per 0.23-mm² HPF)**

| **Parameter** | **n** | **Spearman ρ** | **95% CI** | **Nominal p** |
| --- | --- | --- | --- | --- |
| Whole-tumor PV absolute | 17 | -0.15 | -0.57 to 0.36 | 0.557 |
| Whole-tumor arterial absolute | 15 | 0.05 | -0.46 to 0.55 | 0.849 |
| Whole-tumor Δ absolute | 15 | -0.37 | -0.78 to 0.23 | 0.177 |
| Solid-component PV absolute | 17 | -0.12 | -0.58 to 0.40 | 0.642 |
| Solid-component arterial absolute | 15 | 0.43 | -0.07 to 0.75 | 0.109 |
| Solid-component Δ absolute | 15 | -0.34 | -0.75 to 0.25 | 0.217 |
| Whole-tumor PV normalized | 17 | 0.05 | -0.45 to 0.58 | 0.852 |
| Whole-tumor arterial normalized | 15 | 0.14 | -0.39 to 0.64 | 0.611 |
| Whole-tumor Δ normalized | 15 | 0.00 | -0.51 to 0.55 | 0.990 |
| Solid-component PV normalized | 17 | 0.07 | -0.46 to 0.54 | 0.801 |
| Solid-component arterial normalized | 15 | 0.48 | -0.04 to 0.85 | 0.069 |
| Solid-component Δ normalized | 15 | -0.23 | -0.63 to 0.26 | 0.404 |

Abbreviations: PV, portal-venous; Δ, portal-venous minus arterial; HPF, high-power field. Normalized iodine values are dimensionless tumor-to-aorta ratios.

Supplementary Figure S1. Bland-Altman plot for portal-venous absolute iodine concentration measured in the solid-component ROI. The solid line indicates mean reader bias and dashed lines indicate the 95% limits of agreement.
